# Simultaneous high-resolution detection of multiple transcripts combined with localization of proteins in whole-mount embryos

**DOI:** 10.1186/s12915-014-0055-7

**Published:** 2014-08-15

**Authors:** Theresa Gross-Thebing, Azadeh Paksa, Erez Raz

**Affiliations:** Institute for Cell Biology, ZMBE, Von-Esmarch-Str 56, 48149 Muenster, Germany

**Keywords:** RNA expression, RNA localization, Zebrafish, WISH, FISH, Immunohistochemistry, Fluorescent protein

## Abstract

**Background:**

Whole-mount *in situ* hybridization (WISH) is a fundamental tool for studying the spatio-temporal expression pattern of RNA molecules in intact embryos and tissues. The available methodologies for detecting mRNAs in embryos rely on enzymatic activities and chemical reactions that generate diffusible products, which are not fixed to the detected RNA, thereby reducing the spatial resolution of the technique. In addition, current WISH techniques are time-consuming and are usually not combined with methods reporting the expression of protein molecules.

**Results:**

The protocol we have developed and present here is based on the RNAscope technology that is currently employed on formalin-fixed, paraffin-embedded and frozen tissue sections for research and clinical applications. By using zebrafish embryos as an example, we provide a robust and rapid method that allows the simultaneous visualization of multiple transcripts, demonstrated here for three different RNA molecules. The optimized procedure allows the preservation of embryo integrity, while exhibiting excellent signal-to-noise ratios. Employing this method thus allows the determination of the spatial expression pattern and subcellular localization of multiple RNA molecules relative to each other at high resolution, in the three-dimensional context of the developing embryo or tissue under investigation. Lastly, we show that this method preserves the function of fluorescent proteins that are expressed in specific cells or cellular organelles and conserves antigenicity, allowing protein detection using antibodies.

**Conclusions:**

By fine-tuning the RNAscope technology, we have successfully redesigned the protocol to be compatible with whole-mount embryo samples. Using this robust method for zebrafish and extending it to other organisms would have a strong impact on research in developmental, molecular and cell biology. Of similar significance would be the adaptation of the method to whole-mount clinical samples. Such a protocol would contribute to biomedical research and clinical diagnostics by providing information regarding the three-dimensional expression pattern of clinical markers.

**Electronic supplementary material:**

The online version of this article (doi:10.1186/s12915-014-0055-7) contains supplementary material, which is available to authorized users.

## Background

Whole-mount *in situ* hybridization (WISH) is used to study the RNA expression pattern of genes in the context of tissues in which the RNAs function [1,2]. Chromogenic *in situ* hybridization (ISH) techniques are commonly used in different model organisms for this purpose and are based on an enzymatic reaction that converts a colorless substrate into a dark visible precipitate. Employing chromogenic ISH, up to three different mRNAs can be detected simultaneously in *Drosophila* embryos [3,4]. However, the detection of not more than two different transcripts has thus far been achieved in zebrafish embryos using this method [4].

Compared to chromogenic ISH, fluorescent *in situ* hybridization (FISH) offers a higher resolution and an improved detection of overlapping gene expression patterns in a single sample [5–7]. Combining FISH with confocal microscopy can thus provide spatial information concerning the expression of the investigated RNA within three-dimensional complex samples such as embryos. The signal generated in FISH relies on the binding of a horse radish peroxidase (HRP)-conjugated antibody to modified ribonucleotides of the probe, followed by tyramide signal amplification (TSA), where HRP converts a tyramide conjugated to a fluorophore into a reactive fluorescent intermediate that covalently binds to nearby amino-acid residues, typically tyrosine. Whereas multicolor FISH can potentially be used for detecting multiple transcripts by employing different fluorescent tyramide substrates, the method is less efficient, particularly for RNAs expressed at low levels [6]. To overcome this drawback of the method, modifications to the original FISH protocol have been put forward that significantly enhance the sensitivity of the assay [7,8]. Nevertheless, as other WISH methods, this protocol also relies on the generation of diffusible products that potentially decrease resolution of transcript localization.

**Figure 1:**
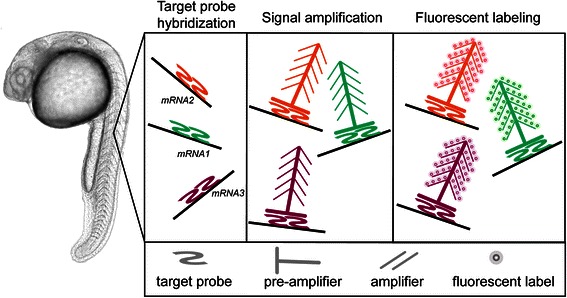
**The RNAscope principle.** Target probe hybridization: Sets of oligonucleotide probes specific for different mRNAs are designed, such that each pair of two z-shaped probes (each approximately 20 bp) hybridizes to the specific target mRNA. In total, 20 pairs are designed for each RNA of interest. In addition to the complementary RNA sequence, each probe contains a spacer, as well as a tail sequence to which the pre-amplifier structure binds in the next step. Signal amplification: As a result of pre-amplifier interaction with the tail sequence, a tree-like scaffold that provides multiple binding sites for fluorescent labels is formed. Fluorescent labeling: Subsequently, distinct fluorescent labels are simultaneously added. Together, the large number of fluorescent labels bound to each target probe pair provides a strong signal, allowing detection of rare mRNA species.

A newly developed alternative method has been reported that allows the simultaneous fluorescent labeling of up to five transcripts by employing an orthogonal amplification with hybridization chain reactions (HCRs) [9]. In this method, a probe set of one to nine probe species is used to target each mRNA molecule. Following probe hybridization, fluorescent RNA hairpins self-assemble into fluorescent amplification polymers [9]. More recently, a novel technology called RNAscope has been developed for RNA detection *in situ.* This method is based on fluorescent signal amplification upon target probe hybridization [10] (Figure 1), and unlike other RNA ISH methods including HCR, it is a rapid protocol that can be completed within less than 2 days. Importantly, as a result of innovative probe design and a detection strategy that relies on non-diffusible fluorogenic products, this method allows the detection of rare transcripts at high resolution, while generating a low background signal [11–14]. In this method, special probes are designed such that they hybridize to the target mRNA, with each probe containing a different tail sequence that provides the base for the assembly of a signal-amplifying scaffold. As this scaffold will only form on a pair of flanking probes, signal specificity is dramatically increased (Figure 1). This method allows the simultaneous detection of up to four mRNA species in formalin-fixed paraffin-embedded cancer cell lines and tumor tissue sections using either fluorescent or chromogenic detection [10]. Thus far, RNAscope has been used in medical research and for detection of histopathological biomarkers in clinical diagnosis [11–15]. As a sensitive and specific assay with multiplex capabilities, RNAscope allows quantitative measurements of RNA levels in the context of the tissue [11] as well as reliable detection of low abundance RNA molecules by flow cytometry [16]. The introduction of relatively small probes prior to the generation of immobile amplification products can contribute to good penetration into the embryo and efficient labeling of rare transcripts.

Whereas RNAscope was successfully employed for sectioned material, determination of RNA expression patterns in larger biological samples, such as intact embryos, would be extremely beneficial in biomedical research and developmental biology and when analyzing large clinical samples. Adapting this method for intact embryos would allow simultaneous high-resolution analysis of developmentally important genes and provide detailed quantitative information concerning expression patterns and expression levels within the developing embryo and tissue.

Based on the RNAscope *ISH* principle, we provide a fine-tuned protocol that we developed for large tissue samples, as exemplified here by using whole-mount zebrafish embryos. The devised protocol includes steps promoting effective preservation of embryo integrity, while allowing efficient penetration of the probes and an excellent signal-to-noise ratio. It is a rapid and robust protocol that facilitates the simultaneous quantitative detection of multiple transcripts within an embryo. Importantly, in addition to revealing the spatial distribution of RNA molecules, the protocol allows the simultaneous determination of protein expression patterns and protein subcellular localization.

## Results and discussion

### General considerations when employing the RNAscope method using embryos

Whereas the RNAscope signal amplification method can be used on tissue sections, a different protocol had to be devised for whole-mount embryonic samples. This conclusion was initially based on our finding that embryos disintegrated when subjected to the protocol designed for staining thin sections [10] (Additional file 1A). While the RNA expression pattern of *myoD*, a universal marker for myogenic cells [17], appeared to be correct (Additional file 1D, arrowhead) in a small proportion of the embryos that withstood the procedure (Additional file 1B), a significant background signal in the notochord was present in both channels, *myoD* and *dapB*, a bacterial mRNA that serves as a negative control [18] (Additional file 1C,D, arrows). Furthermore, following the original protocol, 4’ ,6-diamidino-2-phenylindole (DAPI) staining for visualizing the cell nuclei [19] proved inefficient, presumably due to limited tissue penetration (Additional file 1E). Last, a high background signal was observed in the yolk in all channels (Additional file 1C,D, stars).

Given the shortcomings described above, we sought to optimize the procedure and incubation conditions such that embryo integrity is preserved, while enhancing tissue penetration of reagents and maintaining a low background signal (see Table 1 for a short protocol, Table 2 for reagents, Additional file 2 for a detailed version of the procedure, Additional file 3 for troubleshooting, Table 3 for the probes and Table 4 for label probe combinations and filter set specifications).

**Table 1:** **RNAscope protocol for zebrafish embryos at a glance**

**Step**	**Solution**	**Temperature**	**Time**
**1. Drying**	–	RT	30 min
**2. Digestion**	2 drops of Pretreat 3	RT	20 min
**3. Stop digestion**	PBT 1 ml	RT	Quick 3×
**4. Probe hybridization**	Channel 1: Channel 2: Channel 3 (50:1:1) 50–100 μl/tube	40°C	O/N
**5. Wash**	0.2× SSCT 1 ml	RT	3 × 15 min
**6. Post-fixation**	4% PFA in PBS 1 ml	RT	10 min
**7. Wash**	0.2× SSCT 1 ml	RT	3 × 15 min
**8. Pre-amplifier hybridization**	2 drops of Amp1	40°C	30 min
**9. Wash**	0.2× SSCT 1 ml	RT	3 × 15 min
**10. Signal enhancement**	2 drops of Amp2	40°C	15 min
**11. Wash**	0.2× SSCT 1 ml	RT	3 × 15 min
**12. Amplifier hybridization**	2 drops of Amp3	40°C	30 min
**13. Wash**	0.2× SSCT 1 ml	RT	3 × 15 min
**14. Label**	2 drops of Amp4	40°C	15 min
**15. Wash**	0.2× SSCT 1 ml	RT	3 × 15 min
**16. Counter stain**	2 drops of DAPI or Hoechst: SSCT (1:10000)	4°C	O/N
**17. Mounting**	1% LMP	RT	5–10 min
**18. Microscopy**	–	–	–

DAPI, 4’ ,6-diamidino-2-phenylindole; LMP, low melting point agarose; O/N, overnight; PBS, phosphate-buffered saline; PBT, PBS buffer + 0.01% Tween-20; PFA, paraformaldehyde; RT, room temperature; SSCT, saline-sodium citrate buffer + 0.01% Tween-20.

**Table 2:** **Reagents**

**Reagents**	**Composition**	**Provider**
Danieau’s solution	17.4 mM NaCl, 0.21 mM KCl, 0.12 mM MgSO_4_•7H_2_O, 0.18 mM Ca(NO_3_)_2_, 1.5 mM Hepes	
Fixation solution	4% PFA in PBS, pH 7	
PBS	137 mM NaCl, 2.7 mM KCL, 10 mM Na_2_HPO_4_, 1.8 mM KH_2_PO_4_, pH 7.4	
PBT 0.1%/0.01%	0.1%/0.01% Tween-20 in PBS	
SSCT 0.01%	0.01% Tween-20, 15 mM NaCl, 1.5 mM TriNaCitratdihydrate, pH 7	
Negative control *dapB* (cat.# 310043)	Channel 1, ready-to-use solution	Advanced Cell Diagnostics (ACD), Hayward, CA, USA
Target probe *cxcl12a* (cat.# 406481)	Channel 1: ready-to-use solution	ACD, Hayward, CA, USA
Target probe *myoD* (cat.# 402461-C2)	Channel 2: 50× solution	ACD, Hayward, CA, USA
Target probe *vasa* (cat.# 407271-C3)	Channel 3: 50× solution	ACD, Hayward, CA, USA
Target probe *cxcr4b* (cat.# 418121-C3)	Channel 3: 50× solution	ACD, Hayward, CA, USA
Target probe *egfp* (cat.# 400281)	Channel 1: ready-to-use solution	ACD, Hayward, CA, USA
Target probe *nanos* (cat.# 404521-C2)	Channel 2: 50× solution	ACD, Hayward, CA, USA
Pretreat 3 in RNAscope pretreatment kit (cat.# 320842)	Pretreat 3 ready-to-use solution	ACD, Hayward, CA, USA
RNAscope fluorescent multiplex detection kit (cat.# 320851)	Amp1, Amp2, Amp3, Amp4 AltA, Amp4 AltB, Amp4 AltC, Pretreat 3, DAPI ready-to-use solutions, Wash buffer provided in the kit is not used and is replaced with 0.2× SSCT (0.01% Tween-20)	ACD, Hayward, CA, USA
1% or 4% LMP	1% or 4% low melting point agarose in Danieau’s solution	Invitrogen, Paisley, UK
Hoechst 33342 (cat.# H1399)	Use at a 1:10000 dilution in 0.2× SSCT (0.01% Tween-20)	Invitrogen, Paisley, UK

PBS, phosphate-buffered saline; PBT, PBS buffer + Tween-20; PFA, paraformaldehyde; SSCT, saline-sodium citrate buffer + 0.01% Tween-20.

**Table 3:** **Probes**

**Probe**	**Catalog**	**Accession**	**Selected region**
*dapB*	310043	EF191515	414–862
*Vasa*	407271-C3	NM_131057.1	503–1520
*myoD*	402461-C2	NM_131262.2	2–1083
*cxcl12a*	406481	NM_178307.2	45–1481
*cxcr4b*	418121-C3	NM_131834	2–1048
*egfp*	400281	U55763.1	16–740
*nanos*	404521-C2	NM_131878.1	26–1074

**Table 4:** **Label probe combinations and filter set specifications for microscopy**

**Label**	**Channel 1 probe**		**Channel 2 probe**		**Channel 3 probe**	
	**Exc**	**Emi**	**Exc**	**Emi**	**Exc**	**Emi**
**Amp4 AltA**	495 nm	520 nm	555 nm	575 nm	645 nm	670 nm
**Amp4 AltB**	555 nm	575 nm	495 nm	520 nm	645 nm	670 nm
**Amp4 AltC**	555 nm	575 nm	645 nm	670 nm	495 nm	520 nm

Exc, Excitation; Emi, Emission.

### Increasing embryo integrity

As a first step in establishing the protocol, we modified the composition of the solutions such that the method preserves embryo integrity, while allowing penetration of the relevant reagents. A likely source for embryo damage during washing steps when employing the original RNAscope protocol (Additional file 1) is the inclusion of lithium dodecyl sulfate in the hybridization, amplification and wash buffers. Indeed, using 0.2× SSCT buffer (saline-sodium citrate buffer + 0.01% Tween-20) or 1× PBT (phosphate buffer + 0.01% Tween-20) instead of the original wash buffer resulted in good preservation of the embryos. An additional key factor contributing to embryo preservation was the fixation step. For example, fixing 20-hours post fertilization (hpf) embryos using 4% paraformaldehyde (PFA) in PBS for 1 hour at room temperature (RT) yielded the best results with respect to high signal level and low background, while maintaining tissue integrity. Shorter fixation duration (e.g., 30 min) for 20-hpf embryos resulted in dissociation of the embryos, while such short fixation times were compatible with 24-hpf or older embryos. Considering the importance of the fixation conditions for embryo preservation, we included an additional fixation step (post-fixation, Table 1, point 6) following hybridization of the probes (Table 1, point 4).

The initial embryo-drying step (Table 1, point 1) also appeared to be a crucial factor in preserving the integrity of embryos (Additional file 3). We found that air-drying the embryos for 30 min after methanol (MeOH) removal, followed by digestion with Pretreat solution for 20 min yielded the best results. A combination of both fixation steps and the use of 0.2× SSCT as a wash buffer resulted in optimal preservation of embryos, whose general morphology following RNAscope appears to be similar to that of fixed embryos prior to the procedure (e.g., Additional file 4).

### Background reduction and signal enhancement

A critical issue in methods designed to detect RNA expression patterns in tissues is the achievement of a high signal-to-noise ratio. Three sources could potentially increase background noise in the course of RNAscope: (i) non-specific signal amplification attributed to the amplifiers and subsequent labels not associated with the specific probes, (ii) signal detection due to non-specific hybridization of the probes and (iii) autofluorescence of the tissue.

To examine the effect of the hybridization temperature on the procedure (point (ii) above), we determined the consequences of altering this parameter on the signal and the background levels using *vasa* mRNA, a primordial germ cell (PGC) marker [20]. A complete lack of signal was observed when employing the standard 65°C hybridization temperature used in FISH experiments with zebrafish [6]. Conducting RNAscope using a 55°C or 60°C hybridization temperature resulted in high background or low specific signal (Additional file 5A,B, respectively). Thus, hybridization of the embryos at 50°C and 40°C (the standard temperature of the original RNAscope protocol) was associated with high specific signal and low background (Additional file 5C,D). To reduce the accumulation of excess probes or unbound amplification scaffold in the tissue (points (i) and (ii) above), we extended the washing incubations to three cycles of 15-min washes, which efficiently eliminated the background signal in the notochord. Employing these optimization steps reduced the background, as seen by the lack of a significant signal when using probes directed against the bacterial *dapB* RNA (Additional file 5E). As judged by omitting the fluorescent label from the protocol, the tissue autofluorescence generated in the course of the procedure is low (Additional file 5F).

Interestingly, the fixation step was found to be important not only for preservation of embryo integrity, but for reducing the background signal as well. A possible explanation is that the amplification scaffolds could accumulate in samples that were subjected to non-optimal fixation conditions. In this analysis we found that reducing the fixation time to 30 min at RT for 24-hpf embryos significantly enhanced the specific signal, while reducing the non-specific one (Additional file 5H). Fixation of the same-stage embryos for 24 hours at 4°C resulted in increased background signal (Additional file 5G, star and arrow), while including 0.5 to 2.5% glutaraldehyde in the fixation solution led to a complete elimination of the signal.

To enhance the signal in the thick embryo samples, we prolonged the duration of the hybridization from 2 hours in the original protocol (Additional file 5K) to overnight (O/N), which indeed yielded an enhanced specific signal (Additional file 5L). Employing probes already used once, a specific but weaker signal was detected (Additional file 5J) compared to the results obtained using fresh probes (Additional file 5I). While the original 30-s DAPI treatment [19] led to nuclear staining of the outer cell layers of the embryo (Additional file 1E), O/N incubation at 4°C allowed DAPI to penetrate also into deeper cell layers to visualize the tissue context (Table 1, point 16). Counterstaining the DNA using Hoechst stain provided results similar to those obtained using the DAPI stain. In summary, we found that fixation, hybridization and washing conditions are critical for achieving an optimal signal-to-noise ratio (Additional file 6) and defined the conditions needed to detect the localization of RNA molecules reliably using the RNAscope technology in whole-mount samples.

### Comparing RNAscope-based whole-mount *in situ* hybridization with current *in situ* hybridization methods

**Figure 2:**
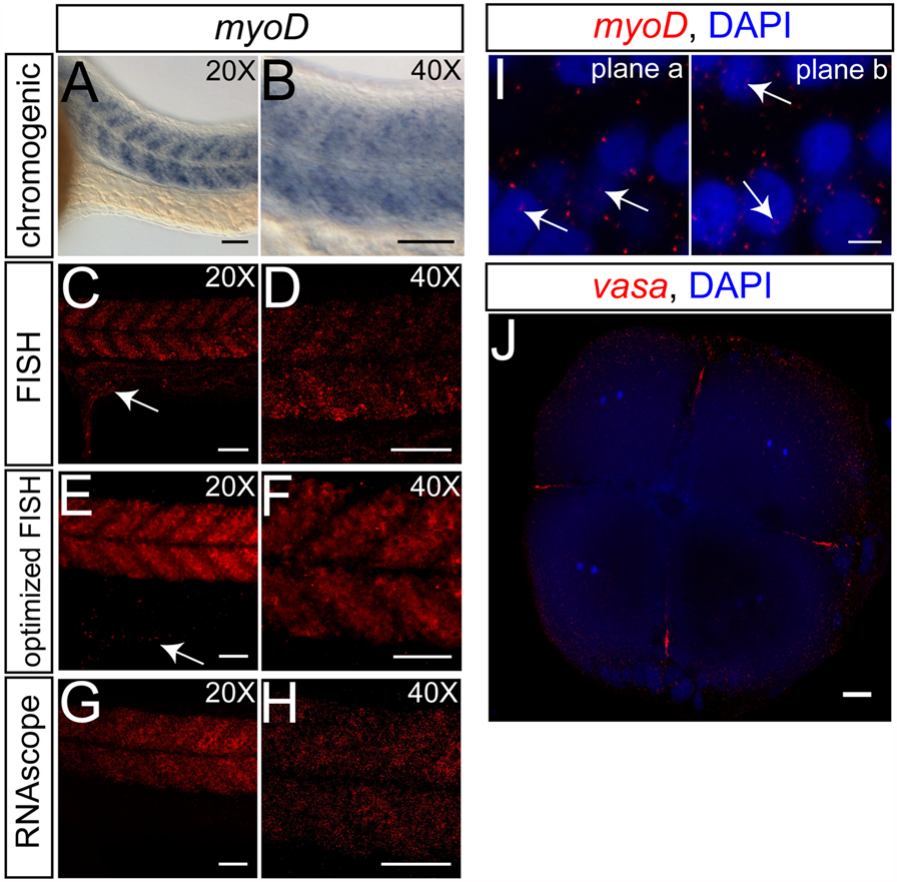
**Comparison of conventional ISH methods with RNAscope.** Using the *myoD* antisense probe to label muscle tissue, whole-mount 24-hpf zebrafish embryos were subjected to chromogenic ISH **(A, B)**, FISH **(C–F)** (arrows in **(C)** and **(E)** indicate non-specific background in the yolk extension) and RNAscope-based mRNA detection **(G, H)**. FISH was either performed using the standard **(C, D)** or optimized conditions **(E, F)**. For panels **(A)**–**(H)**, anterior is to the left and dorsal up. Images were captured using the indicated objectives. **(I)** *myoD* mRNA is detected as distinct spots (shown in red) in the nuclei (arrows) and in the cytoplasm of the cells. Two different optical sections of the same sample from Additional file 7 are displayed. **(J)** The localization of *vasa* RNA in the cleavage furrows of a four-cell stage embryo as visualized using the RNAscope technology (red), with the nuclei stained by DAPI (blue). Scale bars for **(A)**–**(H)** and **(J)**: 50 μm and for **(I)**: 5 μm. Confocal images of whole-mount zebrafish embryos were captured in 3-μm intervals and processed either as z-projections (for **(A)**–**(H)**, and **(J)**) or as different focal planes (for **(I)**) using the ImageJ software. Images **(A)**–**(H)** were captured and subsequently processed using conditions for achieving the best image for each method.

Both chromogenic WISH and FISH are commonly used to study the expression pattern of transcripts in cells and tissues. To compare the RNAscope-based procedure described here with existing ISH methods, we performed chromogenic WISH, whole-mount FISH and RNAscope WISH on 24-hpf zebrafish embryos using the *myoD* probe (Figure 2A–H). Specific expression of *myoD* can be detected in morphologically intact embryos using the chromogenic WISH method (Figure 2A). However, high-resolution imaging of the *myoD* expression revealed its limitations (Figure 2B), as the colored precipitate increased the opacity of the tissue and therefore interfered with imaging of deeper parts of the tissue. On the other hand, expression of *myoD* could be efficiently visualized at higher resolution when whole-mount FISH was employed (Figure 2C,D). Unlike the precipitates seen for chromogenic WISH, employing FISH for zebrafish embryos gave less diffusion of the label, thereby increasing the resolution of the assay and hence, enabling the detection of overlapping expression domains. In spite of the potential for simultaneous detection of multiple transcripts using FISH, the standard procedure appears to be limited to highly expressed RNA molecules [6] and generated non-specific staining in our assay (arrow in Figure 2C).

In addition to the protocol employed above, we examined an optimized whole-mount FISH protocol for zebrafish embryos using a bench-made TSA substrate, permitting higher signal intensities and visualization of less abundant transcripts [8,21]. In comparison to the results obtained using standard FISH, this protocol resulted in a brighter signal for *myoD* mRNA (Figure 2E,F), coupled with reduced background signal from the yolk (Figure 2E, arrow).

Employing the optimized RNAscope-based procedure, specific, high-resolution detection of *myoD* mRNA was achieved (Figure 2G,H). The staining obtained using the RNAscope method appeared to be more distinct and to reflect more accurately the subcellular localization of the transcripts (Figure 2H), compared to the staining in the standard and the optimized whole-mount FISH, which appears more aggregated (Figure 2D) and more diffuse (Figure 2F). The RNAscope method thus allows clear subcellular detection of RNA spots within the cytoplasm and the nuclei of zebrafish embryos (see Figure 2I and Additional file 7 for *myoD* RNA). To further demonstrate the capacity of the RNAscope technique to probe subcellularly localized RNAs, we detected *vasa* mRNA in four-cell stage embryos, a stage when the transcript is confined to the cleavage furrows (Figure 2J).

We have shown that RNAscope is an equivalent alternative to the optimized FISH technique, while presenting several important advantages, in particular with respect to speed and ease of use coupled with high resolution and a low background signal.

### Multicolor quantitative detection of mRNAs in whole-mount zebrafish embryos

**Figure 3:**
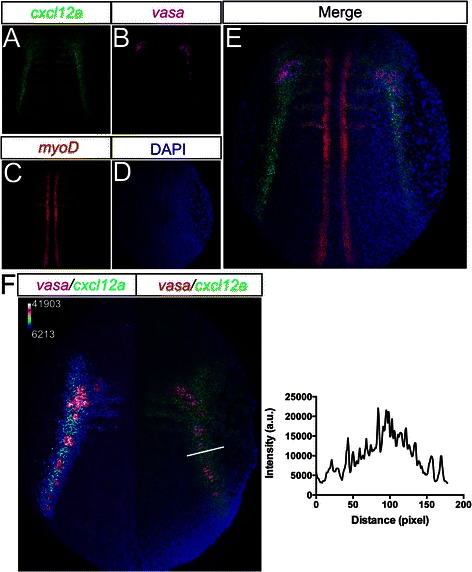
**Simultaneous quantitative detection of different mRNAs in whole embryos.** *cxcl12a* expression pattern **(A)** and *vasa* transcript **(B)** in PGCs are specifically detected in 12-hpf zebrafish embryos. *myoD* RNA expression can be detected in the myotomes of the same sample **(C)** and cell nuclei are stained with DAPI **(D)**. **(E)** An overlay of all the channels. **(F)** 12-hpf zebrafish embryo expressing *cxcl12a* and *vasa* transcripts. The left side of the embryo has the *cxcl12a* expression pattern in pseudocolors together with PGCs in magenta, demonstrating the arrival of the PGCs at domains of high *cxcl12a* expression. The calibration bar is shown for the left half of the embryo. The right side of the embryo shows *cxcl12a* expression domains in green, with the PGCs expressing *vasa* in red and nuclei stained in blue. The intensity profile of *cxcl12a* expression along the white line is presented in the graph on the right in arbitrary units (a.u.). Confocal images of whole-mount zebrafish embryos were captured using a 20× objective and 0.6× digital zoom in 3-μm intervals and subsequently processed as z-projections using the ImageJ software.

We next sought to determine whether the RNAscope technology would allow the detection of multiple transcripts simultaneously in the three-dimensional context of zebrafish embryos. In principle, up to four distinct mRNA molecules can be probed using this technique and thus reveal the spatio-temporal distribution of several mRNA molecules in a single sample. As shown in Figure 3, employing the fine-tuned protocol, *cxcl12a,* an RNA encoding for a chemoattractant for PGCs [22], is specifically detected at a high resolution in 12-hpf embryos (Figure 3A). *vasa*-expressing PGCs (Figure 3B) are positioned within the *cxcl12a* expression domains [22] (Figure 3E), with the developing somites labeled with the *myoD* probe (Figure 3C). In this sample, we have thus successfully visualized transcripts of *cxcl12a, vasa*, *myoD* and DAPI-stained nuclei (Figure 3A–D, respectively). Importantly, the RNAscope technology allows the quantification of the RNA level, by measuring the fluorescence signal emitted by the tissue. This enables the RNA expression level to be represented using pseudocolors, correlating cell behavior with signal distribution (e.g. PGCs arriving at subdomains where *cxcl12a* is highly expressed in the left half of the embryo, as shown in Figure 3F), or quantifying the signal distribution within the *cxcl12*a expression domain (the graph in Figure 3F shows the signal values along the white line). The simultaneous and high-resolution quantitative detection of multiple mRNA molecules in whole embryos constitutes an important tool that is particularly useful in developmental and cell biology.

**Figure 4:**
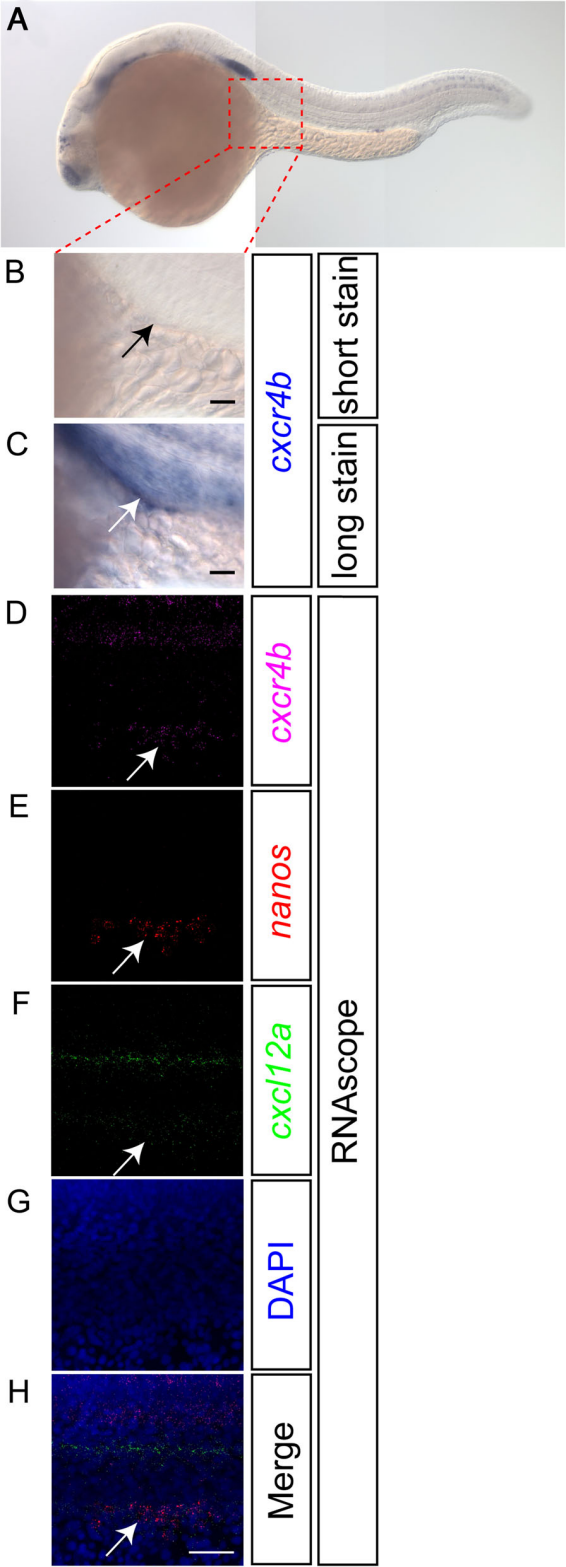
**Detection of rare transcripts with RNAscope.** Using chromogenic ISH, 24-hpf embryos were stained for *cxcr4b* mRNA for short (2 hours at RT) **(A, B)** or long (1 day at RT) **(C)** staining durations. After a short staining reaction, the expression of *cxcr4b* is not detected in the PGCs (**B**, arrow). Prolonged staining of the embryos resulted in weak staining in the PGCs (**C**, arrow). Using the RNAscope protocol with 24-hpf embryos, the weak *cxcr4b* expression in the gonad region (**D**, arrow) was detected in the PGCs that also expressed the *nanos* transcript (**E**, arrow). **(F)** Weak expression of *cxcl12a* in the region of the PGCs is observed as well as strong expression along the lateral line of the embryo. An overlay image of panels **(D)**–**(G)** is shown in **(H)**. Scale bars in **(B)** and **(C)** correspond to 200 μm and that in **(H)** is for **(D)**–**(H)** and signifies 50 μm. For all panels, anterior is left and dorsal up. Confocal images were captured at 3-μm intervals and subsequently processed as z-projections using the ImageJ software.

As the RNAscope technology benefits from special probe design and detection strategy, we examined the detection of rarely expressed transcripts using this technique. *cxcr4b* encodes for the chemokine receptor expressed by PGCs [22], cells of the lateral line primordium and within the nose and hindbrain neurons of a wild-type 24-hpf embryo [23]. Employing chromogenic WISH to detect the RNA of *cxcr4b*, no staining could be observed in PGCs after a short staining reaction (Figure 4A,B). However, extended staining allowed the detection of *cxcr4b* mRNA also in the PGCs [22] (Figure 4C). As an example of a very weakly expressed transcript in PGCs, we thus examined *cxcr4b* expression in these cells in 24-hpf embryos using RNAscope. Indeed, *cxcr4b* mRNA was readily detected in the PGCs in the gonad region (Figure 4D, arrow), as confirmed by the expression of a known PGC marker, *nanos* [24] (Figure 4E,H, arrows). In addition, very weak *cxcl12a* expression can also be detected at 24 hpf in the gonad using RNAscope (Figure 4F, arrow). Thus, the RNAscope technique allows the detection of weakly expressed transcripts in whole-mount zebrafish embryos.

### Simultaneous visualization of RNA and fluorescent proteins

**Figure 5:**
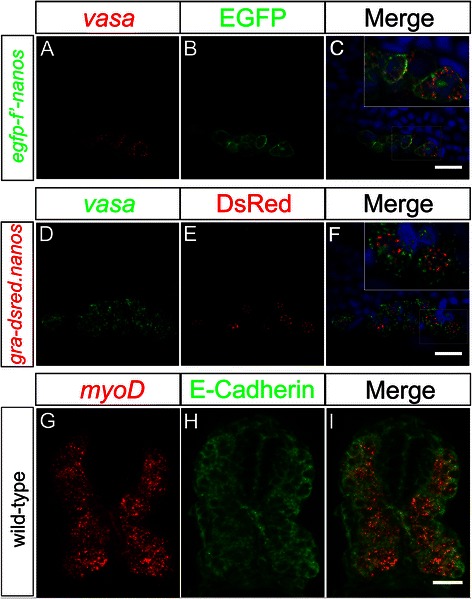
**Detection of protein localization in combination with RNAscope.** Embryos expressing either membrane-bound EGFP in their PGCs **(A, B, C)** or DsRed in their perinuclear germ-cell granules **(D, E, F)** were stained for *vasa* RNA at 20 hpf. *vasa* mRNA expression in the PGCs can be visualized (red in **(A)** and green in **(D)**) together with EGFP and DsRed (**(B)** and **(E)**, respectively). The merged image of panels **(A)** and **(B)** is shown in **(C)** and that of **(D)** and **(E)** in panel **(F)**. Anterior is to the left, dorsal up. A cross section of a 28-hpf wild-type zebrafish embryo showing the *myoD* transcript distribution detected using RNAscope **(G)**, allowing the detection of E-cadherin by antibody staining **(H)**. **(I)** Merged image of the two stainings. Scale bar corresponds to 20 μm. Dorsal is up. Single plane confocal images were captured using a 40× objective and subsequently processed as z-projections using the ImageJ software.

The ability to combine localization of RNA and fluorescent proteins would enable investigation of RNA expression patterns relative to specific embryonic or cellular structures that express fluorescent proteins. We presumed that the relatively low temperature applied in the RNAscope-based protocol (40°C) was likely to preserve the function of fluorescent proteins. We tested this supposition by using 20-hpf zebrafish transgenic embryos, whose PGCs express either EGFP on their plasma membrane or DsRed in the perinuclear structures that characterize PGCs (Figure 5A–F). Indeed, PGCs that expressed *vasa* transcripts in their cytoplasm exhibited a strong EGFP signal on their membrane (Figure 5A,B,C). Similarly, DsRed expressed in PGC granules [25] could be observed along with *vasa* RNA (Figure 5D,E,F), clearly presenting *vasa* localization relative to the granules within PGCs. Increasing the temperature to 50°C, which is compatible with RNA detection, negatively affected the survival of protein fluorescence. We able to show that the RNAscope technology is compatible with immunohistochemistry experiments, where protein localization is determined by use of specific antibodies. As shown in Figure 5G,H,I, we could visualize *myoD* RNA together with E-cadherin labeled by antibodies directed against the protein. The RNAscope method thus allows the simultaneous detection of RNA expression patterns with protein localization using antibodies or fluorescent-protein fusions.

### Penetration of RNAscope target probes into deep tissues of zebrafish embryos

**Figure 6:**
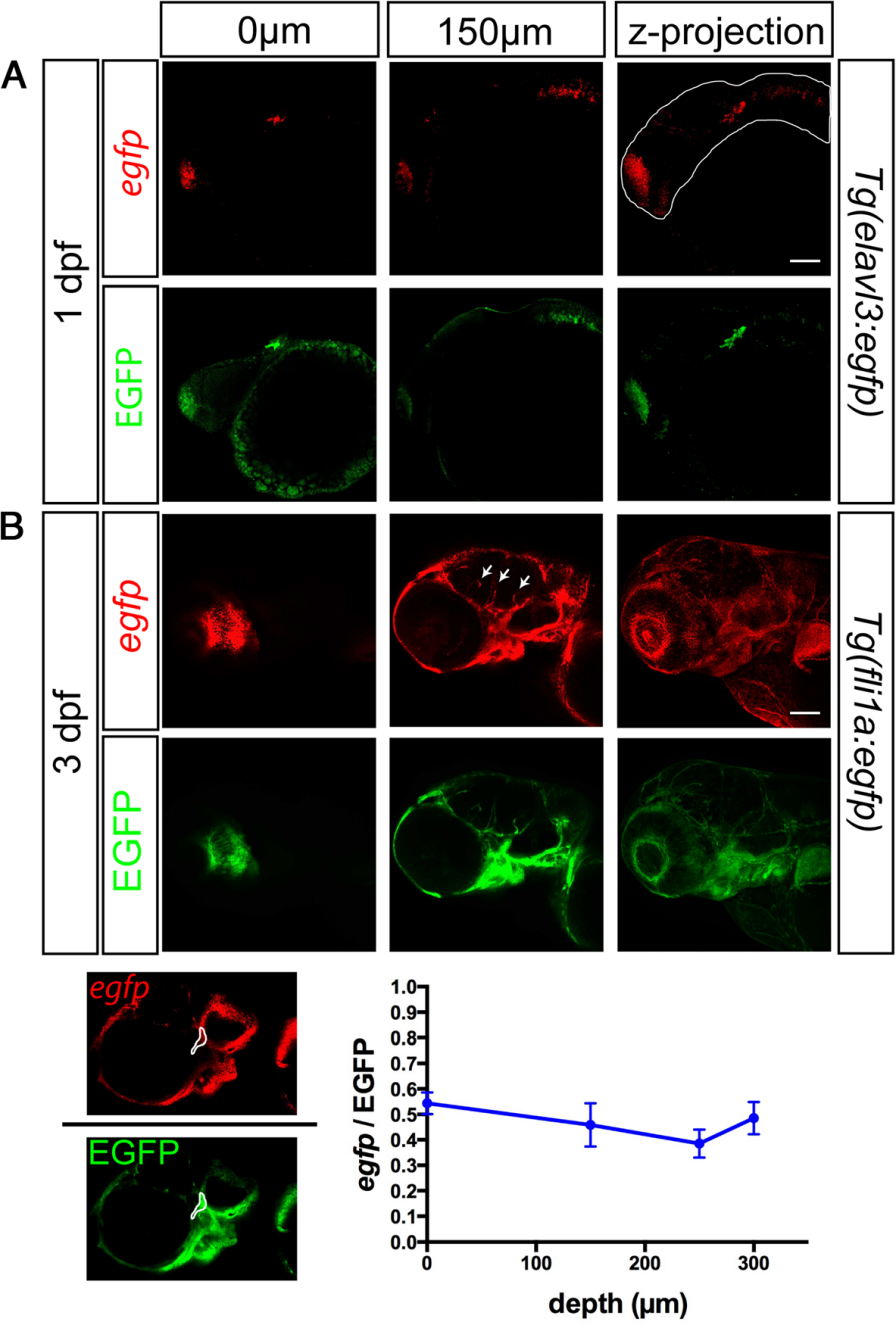
**Labeling of internal tissues by RNAscope probes.** Detection of *egfp* mRNA employing RNAscope in 1-dpf *Tg(elavl3:egfp)* **(A)** (the white line marks the contours of the embryo) and 3-dpf *Tg(fli1a:egfp)* transgenic zebrafish embryos **(B)**. Expression of *egfp* mRNA (red) and endogenous EGFP fluorescence (green) is shown in optical sections of 0 μm and 150 μm depth. The layer labeled as 0 μm was the first one where a signal was detected. The arrows mark internal vessels labeled in RNAscope. A graph showing the signal ratio of *egfp* RNA to EGFP protein fluorescence as measured in central parts of the embryo (an example of a selected area is presented with white outlines) at different depths of the tissue, indicates uniform labeling independent of section depth. Scale bars correspond to 100 μm. Single plane confocal images were captured using a 20× objective and 0.6× digital zoom in 3.5 to 4 μm intervals. Optical sections show merged images of three adjacent confocal planes. Sections were obtained from the lateral aspects of the embryo. Z-projections of all optical sections are shown on the right. Examining the RNA expression in 3-day-old larvae, *egfp* expression is detected in the lens, which does not exhibit EGFP signal, presumably reflecting non-specific staining of this structure. See also Additional files 8 and 9.

As investigating the RNA expression pattern in internal organs of embryos of more advanced developmental stages is often desired, we evaluated the RNAscope method in this context. To this end, we used 1-day post fertilization (dpf) *Tg(elavl3:egfp)* and 3-dpf *Tg(fli1a:egfp)* transgenic embryos that express *egfp* RNA and EGFP protein in neuronal cells [26] or in their vasculature [27], respectively. *egfp* RNA could be detected in internal tissues of 1- and 3-day-old larvae (Figure 6, Additional files 4E and 8). Importantly, consistent with the idea that the 20-bp short probes used in the procedure can readily penetrate the tissue, the signal provided by RNAscope was comparable to that generated by the endogenous EGFP fluorescence (which withstands RNAscope and as such is not limited by reagent penetration) at different tissue depths (Figure 6B). To demonstrate the penetration of RNAscope probes and reagents into deeper tissues of 3-day-old embryos, irrespective of imaging-related constrains, we sectioned the *Tg(fli1a:egfp)* transgenic embryos *following* whole-mount RNAscope. *egfp* RNA could be detected in internal vessels at 170 and 250 μm tissue depths (frontal view, Additional file 9).

The RNAscope method allows the detection of RNA molecules in a wide range of developmental stages and tissue depths in whole-mount zebrafish embryos.

## Conclusions

ISH is a fundamental method for studying gene expression in cells, tissues and whole organisms. Using zebrafish as an example, we present the development of RNAscope-based detection of multiple transcripts in whole-mount embryos, offering a procedure that is compatible with protein localization. The improved protocol preserves embryo integrity and is remarkably rapid. The uniquely designed probes for RNAscope and the amplification of target signals allow the detection of rarely expressed transcripts. In addition, the exclusion of antibodies and the lack of diffusible reaction staining products contributed to increased resolution and excellent signal-to-noise ratios. While not tested in this study, we assume that minor modifications to the final protocol would allow its application to embryos of other species and large tissue samples as well. The implementation of such modifications in whole-mount samples would contribute to biomarker analysis of clinical samples, diagnostics and basic research.

## Methods

### Zebrafish work

Zebrafish (*Danio rerio*) with the AB genetic background and transgenic fish carrying *Tol-kop-egfp-f’-nanos 3′ UTR* [28], *Tol-kop-gra-dsRed-nanos 3’ UTR* [29], *fli1a-egfp* [27] or the *elavl3-egfp* [26] transgene were used. The zebrafish were handled according to the laws of the state of North Rhine-Westphalia, supervised by the veterinarian office of the city of Muenster.

### RNAscope assay

Zebrafish embryos were fixed according to their developmental stage (Additional file 2), in 4% PFA in PBS (137 mM NaCl, 2.7 mM KCl, 10 mM Na_2_HPO_4_, 1.8 mM KH_2_PO_4_, pH 7) at RT prior to (for embryos younger than 24 hpf), or after (for 24-hpf embryos and older) hand dechorionation. For each experimental point, 20 embryos were processed in one 1.5 ml Eppendorf tube. A series of increasing MeOH concentrations (25%, 50%, 75%, 2× 100%) in 0.1% PBT (0.1% Tween-20 in PBS, pH 7.4) was used to dehydrate the embryos stepwise in 5-min washes. After the last MeOH wash, embryos were stored at -20°C for at least one night. The embryos were then air-dried for 30 min at RT and subjected to the RNAscope-based signal amplification (Advanced Cell Diagnostics). Protease digestion of embryos using Pretreat 3 [10] was performed for 20 min at RT followed by rinsing the embryos three times in 0.01% PBT (0.01% Tween-20 in PBS, pH 7.4). Target probe hybridization (hybridization buffer 1: 6× SSC (1× SSC is 0.15 mol/L NaCl, 0.015 mol/L Na-citrate), 25% formamide, 0.2% lithium dodecyl sulfate, blocking reagents [10]) was performed at 40°C O/N to allow the preservation of protein fluorescence. A hybridization temperature of 50°C results in comparably high signal intensities in the sample and can thus be employed for background problems when the activity of fluorescent proteins is not of importance. Following recovery of the probes, the embryos were washed three times for 15 min in 0.2× SSCT (0.01% Tween-20, 3 mM NaCl, 0.3 mM TriNaCitratdihydrate, pH 7) at RT. An additional fixation step was performed using 4% PFA for 10 min at RT.

For RNA detection, incubation with the different amplifier solutions was performed in a water bath at 40°C. The pre-amplifier (2 nmol/L) was in hybridization buffer 2 (20% formamide, 5× SSC, 0.3% lithium dodecyl sulfate, 10% dextran sulfate, blocking reagents). The amplifier (2 nmol/L) was in hybridization buffer 2. The label probe (2 nmol/L) was in hybridization buffer 3 (5× SSC, 0.3% lithium dodecyl sulfate, blocking reagents) [10].

After each hybridization step, the embryos were washed three times with 0.2× SSCT for 15 min. The embryos were then incubated with DAPI ready-to-use solution (Advanced Cell Diagnostics) O/N at 4°C with slow agitation. Prior to imaging, embryos were rinsed in 0.01% PBT, mounted in 1% low melting point agarose (LMP) and imaged in 1× PBS solution.

### Conventional *in situ* hybridization assays

One-color WISH was performed as previously described [30], with modifications according to [1,31]. Whole-mount FISH was performed based on [6] and the optimized FISH according to [8]. Digoxigenin-labeled *myoD* [GenBank:NM_131262] and *cxcr4b* [GenBank:NM_131834] probes were synthetized using T7 or Sp6 polymerase, respectively, according to the manufacturer’s protocol (Roche, Basel, Switzerland).

### Immunohistochemistry

Following the RNAscope protocol, 28-hpf embryos were embedded in 4% LMP and sectioned into 100-μm slices using a vibratome (Leica, Wetzlar, Germany VT1000E). Sections were permeabilized (0.1% Tween-20 and 0.3% Triton-X-100 in PBS) for 1 hour at RT and incubated in blocking buffer (0.3% Triton X-100 and 4% BSA in PBS) O/N at 4°C. Incubation with a mouse monoclonal primary antibody targeting E-cadherin (BD, San Jose, CA, USA Biosciences, 610181) in blocking buffer (1:100) was performed O/N at 4°C followed by three washes for 5 min in 0.3% Triton X-100 in PBS. The secondary antibody (rabbit Alexa 568 anti-mouse IgG, Invitrogen) was applied at 1:1000 dilution in blocking buffer and incubated O/N at 4°C in the dark. The sections were then washed three times, mounted on slides using fluorescence mounting medium (Dako, Hamburg, Germany) and imaged.

### Microscopy and image analysis

Fluorescent images were acquired on an LSM 710 microscope (Zeiss, Oberkochen, Germany). Chromogenic ISH images were obtained on an Axioplan2 epifluorescent microscope (Zeiss, Oberkochen, Germany). Whole-clutch bright-field images were captured using a SteREO Discovery.V12 (Zeiss, Oberkochen, Germany) and AxioCam MRc5 camera (Zeiss, Oberkochen, Germany). Images were processed using ImageJ software (National Institutes of Health) and Imaris (Bitplane AG, Zurich, Switzerland).

Signal intensity measurements to assess RNAscope probe penetration were performed on confocal images after subtracting background levels using the rolling ball algorithm. The measurements were performed at four different selected expression sites in the vessels. Measurements were repeated for three independent embryos. Signal-to-noise ratio was determined employing the ROI manager of ImageJ, dividing the average pixel intensity of an area expressing *egfp* under the control of the *fli1a* promoter with that in a region where *egfp* is not expressed. This procedure was repeated six times in different confocal planes.

## Additional files

Additional file 1:
**The original RNAscope protocol causes embryo damage and non-specific staining. (A, B)** Using the RNAscope multiplex detection protocol for RNA ISH, disintegration of zebrafish embryos was observed (shown for 24-hpf embryos). Under these conditions, detection of RNA using probes against *dapB* (a bacterial RNA not expressed in zebrafish) **(C**) and *myoD* **(D)** was associated with a background signal in the notochord and autofluorescence in the yolk (arrows and stars, respectively). **(E)** Counterstaining with DAPI was not efficient with this protocol. Nevertheless, some specific staining in the developing muscles can be observed (arrowhead in (**D**)).
Additional file 2:
**Detailed RNAscope protocol for detection of transcripts in whole-mount zebrafish embryos.**

Additional file 3:
**Troubleshooting table.**

Additional file 4:
**Morphology of the embryos following RNAscope.** The morphology of fixed embryos before **(A)** and after the RNAscope technique **(B**–**H)** is demonstrated for 24-hpf *Tg(fli1a:egfp)* **(A**–**E)** and 2, 3 and 4-dpf **(F, G, H)** zebrafish embryos, which are not affected by the procedure. **(C)** *egfp* mRNA is detected using RNAscope. **(D)** Counterstaining with DAPI shows that the morphology of the embryos is preserved. **(E)** Overlay of the bright-field and *egfp* probe. The images were captured using a 5× objective.
Additional file 5:
**RNAscope troubleshooting.** Several modifications were applied during the different optimization steps with the RNAscope technique for zebrafish embryos using *vasa* **(A**–**D, G, H)**, *myoD* **(I**–**L)** and *dapB* **(E, F)** probes. Elevation of hybridization temperature to 55°C or 60°C increased the background signal and reduced signal intensities respectively **(A, B)** compared to the optimal hybridization temperature at 50°C **(C)** or 40°C **(D)**. **(E)** Employing the optimized RNAscope protocol using the bacterial *dapB* RNA as a negative control did not show non-specific signal. **(F)** The remaining weak fluorescent signal in the yolk corresponds to autofluorescence as determined by omitting the probe-labeling (Amp4) step in RNAscope. Increased non-specific signal in the myotome and the notochord was observed upon fixation of 1-dpf embryos for 24 hours at 4°C (star and arrow respectively in (**G**)) compared to the fixation for 30 min at RT **(H)**. The *myoD* transcript was detected using probes already used once with 24-hpf embryos **(J)**, albeit with lower intensity signals compared to those obtained with freshly prepared probes **(I)**. Two hours of hybridization did not allow sufficient probe penetration into inner tissues ((**K**), star in the myotome area), while the O/N hybridization showed proper labeling of the myotomes **(L)**. 20-hpf **(A**–**F)** or 24-hpf **(G**–**L)** embryos were used. Scale bar: 50 μm. Anterior to the left; dorsal up. hyb, hybridization.
Additional file 6:
**Signal-to-noise ratio obtained with RNAscope.** Employing the RNAscope protocol on 1-dpf *Tg(fli1a:egfp)* transgenic embryos in which *egfp* transcript was probed, the signal in six different regions expressing *egfp* transcript (red) was divided by that in adjacent areas within the embryo lacking expression (green). s/n, signal-to-noise.
Additional file 7:
**Distinct localization of mRNAs using RNAscope.** *myoD* transcript (red) is detected as highly defined spots at high resolution in the cytoplasm and within the DAPI-labeled nuclei (blue). The movie was processed using the Imaris software.
Additional file 8:
**Labeling of internal tissues by RNAscope probes in 3-dpf zebrafish larvae.** A 3-dpf *Tg(fli1a:egfp)* zebrafish larvae imaged for EGFP expression (left) and *egfp* RNA (right). A non-specific signal for *egfp* was detected in the lens. The movie was processed using the Imaris software. See also Additional file 9.
Additional file 9:
**Detection of internal tissues by RNAscope.** Following RNAscope on whole-mount 3-dpf *Tg(fli1a:egfp)* transgenic samples, the embryos were sectioned (100 μm) and subsequently imaged for *egfp* mRNA (red) and endogenous EGFP fluorescence (green) at 170 μm and 250 μm tissue depths. Scale bars correspond to 100 μm. Single plane confocal images were captured using a 20× objective. Dorsal is up.

## Abbreviations

ACD: Advanced Cell Diagnostics
bp: base pair
BSA: bovine serum albumin
DAPI: 4’,6-diamidino-2-phenylindole
dpf: days post fertilization
FISH: fluorescent *in situ* hybridization
HCR: hybridization chain reaction
hpf: hours post fertilization
HRP: horse radish peroxidase
ISH: *in situ* hybridization
LMP: low melting point agarose
MeOH: methanol
O/N: overnight
PBS: phosphate-buffered saline
PFA: paraformaldehyde
PGC: primordial germ cell
RT: room temperature
TSA: tyramide signal amplification
WISH: whole-mount *in situ* hybridization
