# Supplementary figures and images for: Simultaneous high-resolution detection of multiple transcripts combined with localization of proteins in whole-mount embryos
Source: BMC Biol. 2014 Aug 15;12:55. doi: 10.1186/s12915-014-0055-7 (PMC4172952; doi:10.1186/s12915-014-0055-7)

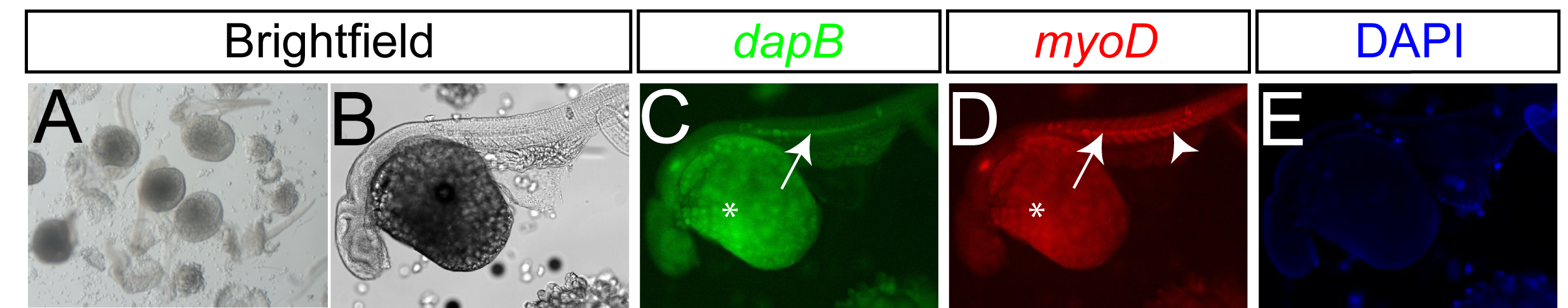

Supplement: Additional file 1: — The original RNAscope protocol causes embryo damage and non-specific staining. (A, B) Using the RNAscope multiplex detection protocol for RNA ISH, disintegration of zebrafish embryos was observed (shown for 24-hpf embryos). Under these conditions, detection of RNA using probes against dapB (a bacterial RNA not expressed in zebrafish) (C) and myoD (D) was associated with a background signal in the notochord and autofluorescence in the yolk (arrows and stars, respectively). (E) Counterstaining with DAPI was not efficient with this protocol. Nevertheless, some specific staining in the developing muscles can be observed (arrowhead in (D)). [file 12915_2014_55_MOESM1_ESM.tiff]

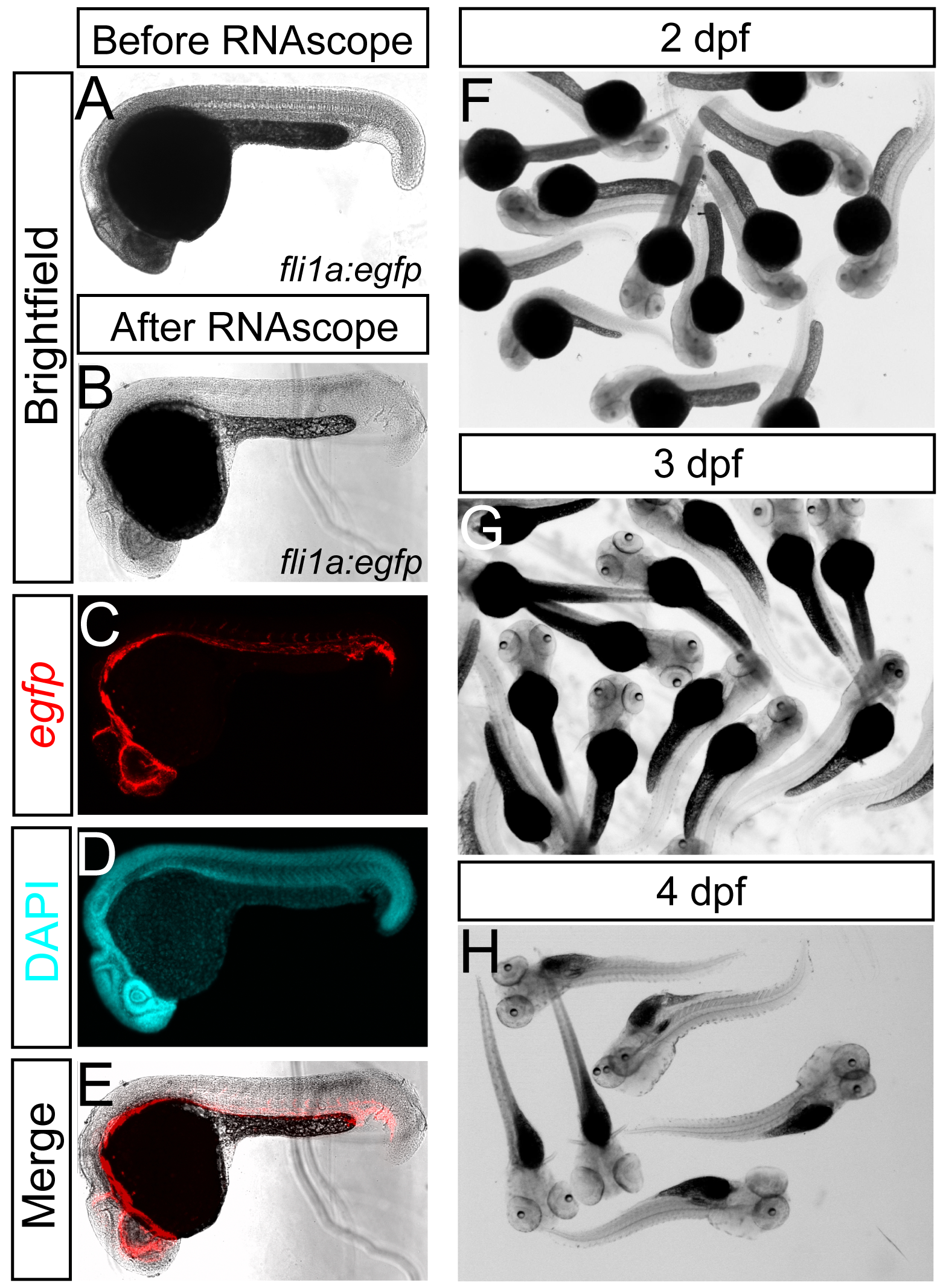

Supplement: Additional file 4: — Morphology of the embryos following RNAscope. The morphology of fixed embryos before (A) and after the RNAscope technique (B–H) is demonstrated for 24-hpf Tg(fli1a:egfp) (A–E) and 2, 3 and 4-dpf (F, G, H) zebrafish embryos, which are not affected by the procedure. (C) egfp mRNA is detected using RNAscope. (D) Counterstaining with DAPI shows that the morphology of the embryos is preserved. (E) Overlay of the bright-field and egfp probe. The images were captured using a 5× objective. [file 12915_2014_55_MOESM4_ESM.tiff]

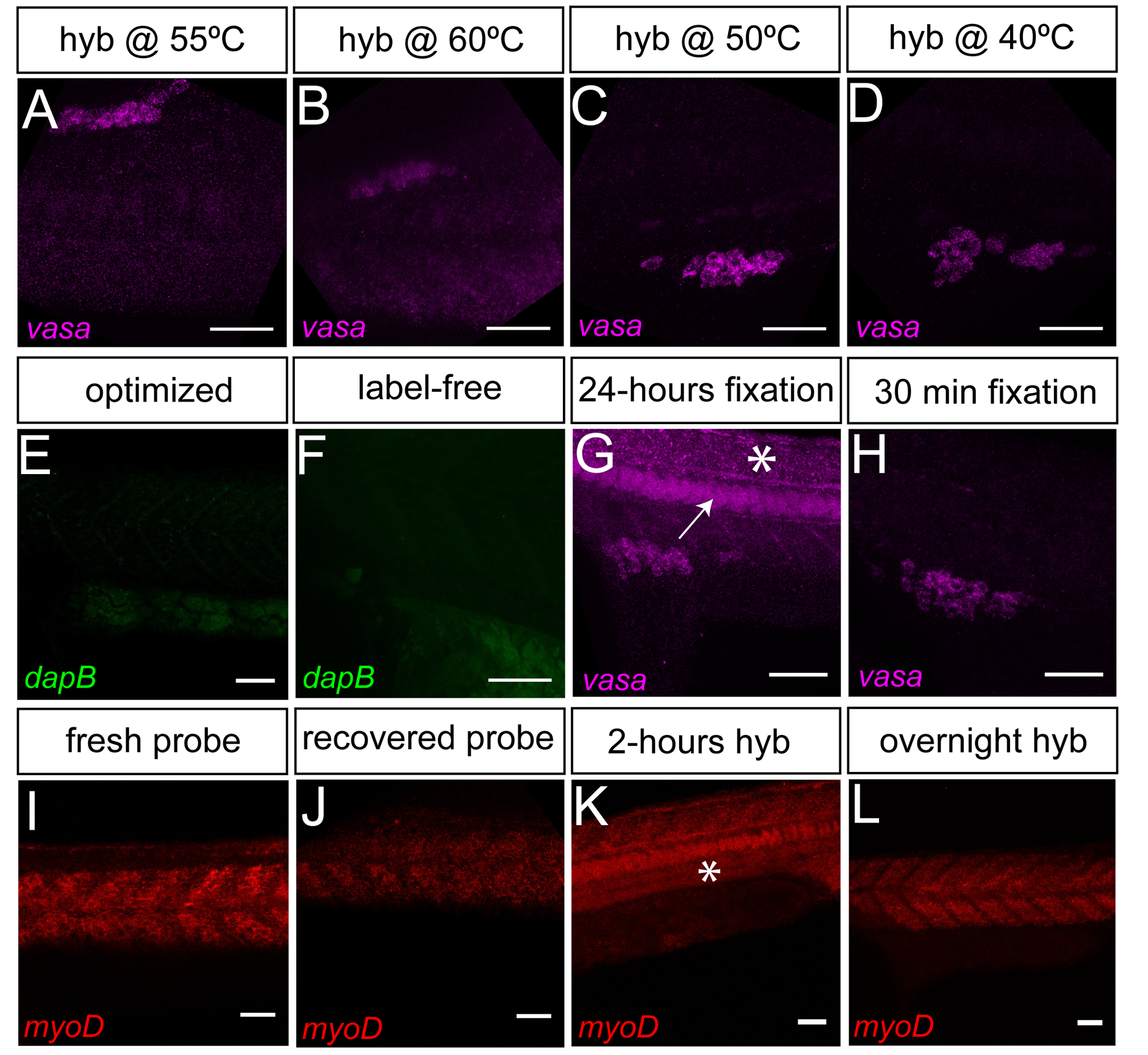

Supplement: Additional file 5: — RNAscope troubleshooting. Several modifications were applied during the different optimization steps with the RNAscope technique for zebrafish embryos using vasa (A–D, G, H), myoD (I–L) and dapB (E, F) probes. Elevation of hybridization temperature to 55°C or 60°C increased the background signal and reduced signal intensities respectively (A, B) compared to the optimal hybridization temperature at 50°C (C) or 40°C (D). (E) Employing the optimized RNAscope protocol using the bacterial dapB RNA as a negative control did not show non-specific signal. (F) The remaining weak fluorescent signal in the yolk corresponds to autofluorescence as determined by omitting the probe-labeling (Amp4) step in RNAscope. Increased non-specific signal in the myotome and the notochord was observed upon fixation of 1-dpf embryos for 24 hours at 4°C (star and arrow respectively in (G)) compared to the fixation for 30 min at RT (H). The myoD transcript was detected using probes already used once with 24-hpf embryos (J), albeit with lower intensity signals compared to those obtained with freshly prepared probes (I). Two hours of hybridization did not allow sufficient probe penetration into inner tissues ((K), star in the myotome area), while the O/N hybridization showed proper labeling of the myotomes (L). 20-hpf (A–F) or 24-hpf (G–L) embryos were used. Scale bar: 50 μm. Anterior to the left; dorsal up. hyb, hybridization. [file 12915_2014_55_MOESM5_ESM.tiff]

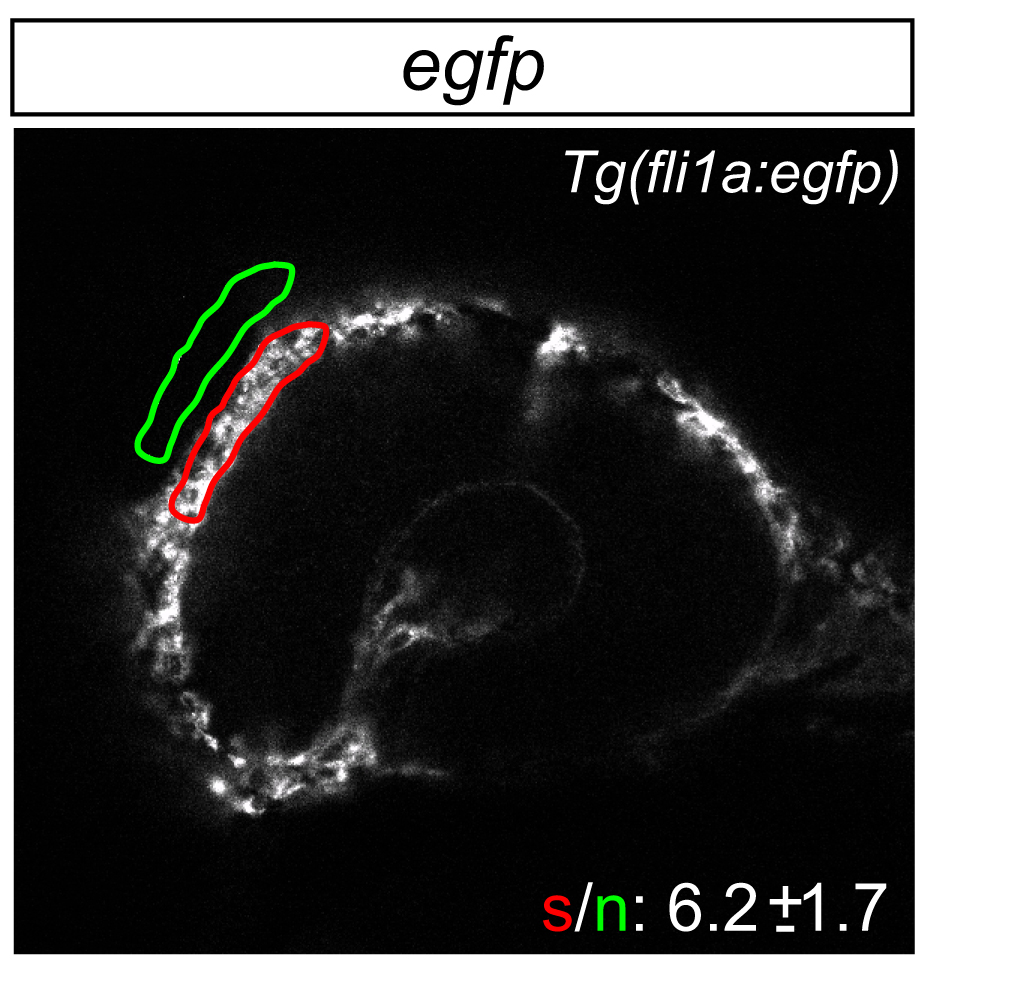

Supplement: Additional file 6: — Signal-to-noise ratio obtained with RNAscope. Employing the RNAscope protocol on 1-dpf Tg(fli1a:egfp) transgenic embryos in which egfp transcript was probed, the signal in six different regions expressing egfp transcript (red) was divided by that in adjacent areas within the embryo lacking expression (green). s/n, signal-to-noise. [file 12915_2014_55_MOESM6_ESM.jpeg]

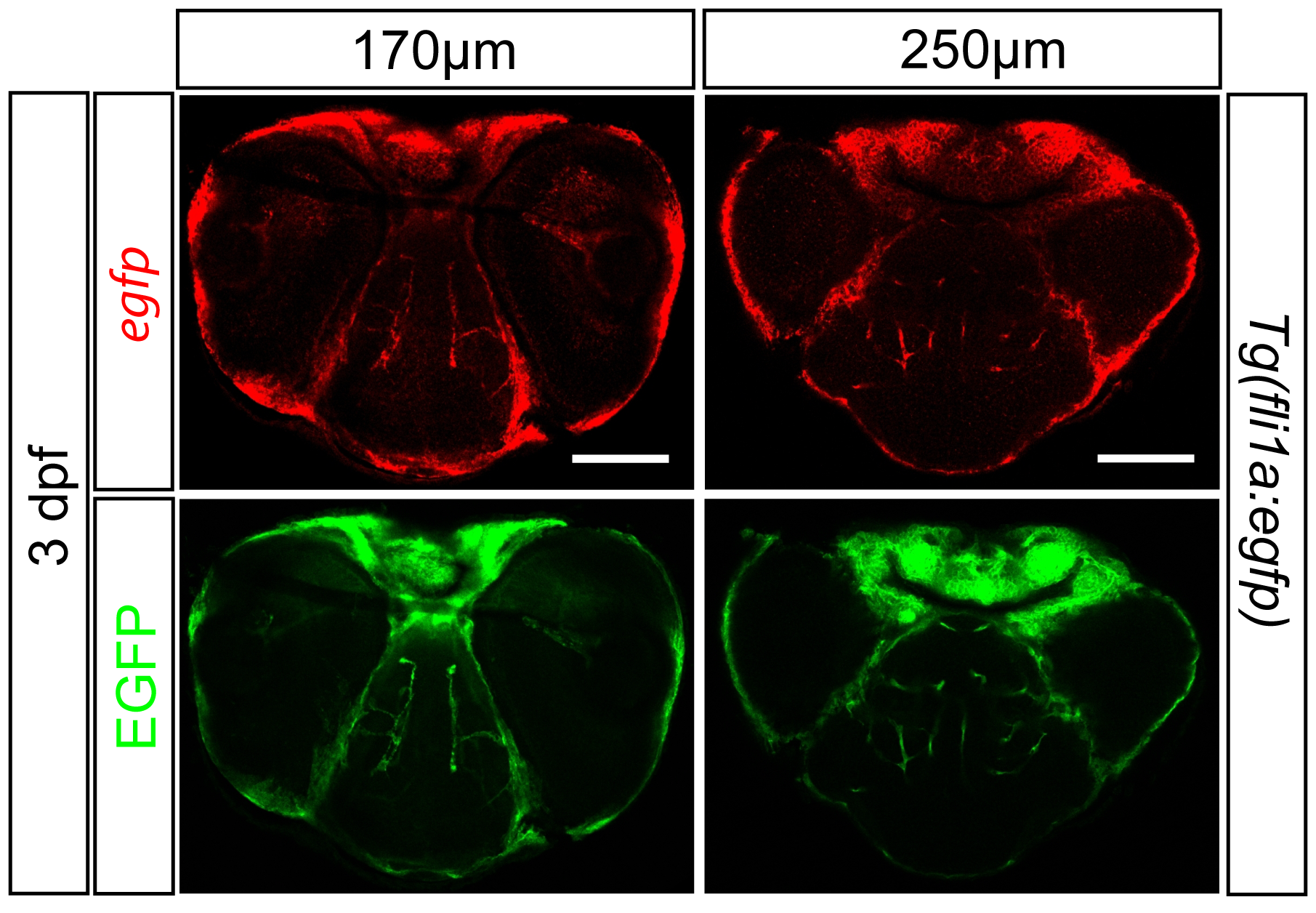

Supplement: Additional file 9: — Detection of internal tissues by RNAscope. Following RNAscope on whole-mount 3-dpf Tg(fli1a:egfp) transgenic samples, the embryos were sectioned (100 μm) and subsequently imaged for egfp mRNA (red) and endogenous EGFP fluorescence (green) at 170 μm and 250 μm tissue depths. Scale bars correspond to 100 μm. Single plane confocal images were captured using a 20× objective. Dorsal is up. [file 12915_2014_55_MOESM9_ESM.tiff]
