# Supplementary material for: Simultaneous high-resolution detection of multiple transcripts combined with localization of proteins in whole-mount embryos
Source: BMC Biol. 2014 Aug 15;12:55. doi: 10.1186/s12915-014-0055-7 (PMC4172952; doi:10.1186/s12915-014-0055-7)
Supplement: Additional file 2: — Detailed RNAscope protocol for detection of transcripts in whole-mount zebrafish embryos. [file 12915_2014_55_MOESM2_ESM.doc]

Additional file 2

This protocol includes a step-by-step procedure for use in the laboratory.

| **Overview** | The RNAscope Multiplex Fluorescent Assay for whole-mount zebrafish embryos can be completed within 2 days, allowing the detection of up to 4 different mRNAs simultaneously in different colors in combination with protein fluorescence. |
| --- | --- |

**Before the assay**

*** Unless otherwise mentioned, 0.01% of Tween concentration is used throughout the protocol (in PBT and 0.2X SSCT buffers).**

| **Fixation** | - Dechorionate embryos manually in PBT (0.1x Tween) in a Petri dish. After dechorionation transfer the embryos to 1.5ml Eppendorf tubes using a glass Pasteur pipette. For embryos younger than 24 hpf, first fix and then dechorionate. 24-hpf embryos and older were fixed without the chorion.      - Fix embryos at RT in 1ml freshly prepared or freshly thawed 4% PFA in PBS with the tube positioned on its side.   Fixation time:  4-cells to 8 hpf: 4 hours  12 to 20 hpf: 1 hour  24 hpf to 4 dpf: 30 minutes  The optimal fixation times for different stages and specific probes can be further optimized if needed.     - Remove the fixation solution and wash 3x5 minutes in 1ml PBT (0.1x Tween) at RT. - Dehydrate embryos through a series of 25%, 50%, 75% MeOH in PBT (0.1x Tween) for 5 min each. - Transfer embryos to 100% MeOH for 5 min, replace it with fresh MeOH and store at -20°C for overnight or longer. |
| --- | --- |
| **Prepare wash buffers** | - Prepare 0.2X SSCT as the main wash buffer. - Prepare 1X PBT. |
| **Heat Water bath** | - Heat the water bath to 40°C. |
| **Mix Target probes** | - Warm the probes at 40°C in the water bath for 10 min to dissolve precipitation and then bring it to RT. - Spin down briefly the C2 and C3 probes to bring down the contents from the cap. - Mix well the target probes of C1, C2 and C3 in a tube at 50:1:1 ratio. Use a final volume of 50-100µl per tube. |
| **Reagents to RT** | - Transfer all reagents from 4°C to RT. |

The RNAscope detection step

| **1. Drying** | - Remove the 100% MeOH completely from the tube of embryos. - Let the embryos air-dry at RT for 30 min. |
| --- | --- |
| **2. Protease digestion** | - Add 2 drops of Pretreat 3 and incubate at RT for 20 min - During the incubation position the tubes horizontally with very slow agitation to ensure homogenous treatment of the embryos. |
| **3. Stop digestion** | - Remove the Pretreat 3 solution. - Rinse the embryos 3x 1ml PBT at RT. |
| **4. Probes hybridization** | **NOTE:**   - Pre-mixed probes should be pre-warmed to 40°C and then cooled down to RT before use. - Add 50-100µl of mixed target probes per tube. - Incubate overnight at 40°C.   In case of high background and no need for fluorescent protein detection, use 50ºC. |
| **5. Probes removal** | - Recover the probes in a new tube. The recovered probes can be reused. - Wash the embryos 3x 15 minutes with 1ml of 0.2X SSCT at RT. |
| **6. Postfixation** | - Fix the embryos again in 1ml of 4% PFA in PBS at RT for 10 min. - Lay the tube on its side. |
| **7. Wash** | - Wash 3x 15 minutes with 1ml 0.2X SSCT at RT. |
| **8. Preamplifier hybridization** | - Remove the SSCT and replace it with 2 drops of Amp1. Gently tap the tube to mix completely. - Incubate the embryos at 40°C for 30 min. |
| **9. Wash** | - Wash 3x 15 minutes with 1ml 0.2X SSCT at RT. |
| **10. Signal enhancement** | - Aspirate the SSCT and add 2 drops of Amp2. Gently tap the tube. - Incubate the embryos at 40°C for 15 min. |
| **11. Wash** | - Wash the embryos 3x 15 minutes with 1ml 0.2X SSCT at RT. |
| **12. Amplifier hybridization** | - Aspirate the SSCT and replace it with 2 drops of Amp3. Tap the tube mildly. - Incubate at 40°C for 30 min. |
| **13. Wash** | - Wash 3x 15 minutes in 1 ml 0.2X SSCT at RT. |
| **14. Label probe hybridization** | - Add 2 drops of Amp4 and tap the tube mildly and incubate at 40°C for 15 min. Using Amp4 alternative solutions (AltA, B or C) different probe and fluorophore combination is possible. (See also materials and methods) |
| **15. Wash** | - Rinse 3x 15 minutes with 1ml 0.2X SSCT at RT. |
| **16. Counter stain** | - Remove the SSCT and add 2 drops of DAPI per tube or Hoechst: 0.2X SSCT at a 1:10000 ratio. - Incubate overnight at 4°C with slow agitation. |
| **17. Preparation for microscopy** | - Rinse the embryo with 1ml 1X PBT and then prepare for imaging using 1% LMP in a Petri dish. - Fill the Petri dish with 1X PBS to allow imaging using water-immersion objectives. |
| **18. Microscopy** | - Image the samples using a fluorescent confocal microscope.   Go to the section of “Label Probe Combination, filter set specification for microscopy ”.   - The embryos do not have to be “deyolked”, since the presented RNAscope procedure produces minimal background fluorescence in the yolk. - Wrap tubes in aluminium foil and store at 4°C for later imaging. |
