# Supplementary material for: Simultaneous high-resolution detection of multiple transcripts combined with localization of proteins in whole-mount embryos
Source: BMC Biol. 2014 Aug 15;12:55. doi: 10.1186/s12915-014-0055-7 (PMC4172952; doi:10.1186/s12915-014-0055-7)
Supplement: Additional file 3: — Troubleshooting table. [file 12915_2014_55_MOESM3_ESM.doc]

Additional file 3

This file contains a trouble-shooting procedure.

When using embryos of other species, or zebrafish embryos of other stages, certain problems might arise, possible solutions to which are provided in this file.

| **Problem** | **Troubleshoot** | **Solution** |
| --- | --- | --- |
| **Dissociation of the embryos** | - Fragile embryos due to insufficient fixation time | - Increase fixation and post-fixation times to enhance the stability of embryos - Carefully handle embryos, do not tap the tube too roughly |
|  | - Harsh buffer conditions of hybridization and Amp solutions slowly reduce the embryo integrity | - Increase fixation times to enhance the integrity of embryos - Carefully handle embryos, do not tap the tubes too roughly - Decrease incubation time in the different Amp solutions and/or hybridization solution |
|  | - Too short time of air-drying after MeOH removal | - Air-dry embryos not for less than 30 min |
|  | - Too long digestion of the embryos in Pretreat3 | - Reduce the Pretreat3 incubation time |
| **Sticking of the embryos together** | - Too much MeOH removed prior to air-drying step | - Leave minimal amounts of MeOH prior to air-drying |
| **Background signal** | - Autofluorescence | - Adjust the fixation time - Use fresh PFA for fixation - Don't use embryos stored for too long |
|  | - Non-specific amplification | - Increase the number of washes and washing duration while subjecting the tubes to very slow agitation - Lay the tubes horizontally to allow efficient washing - Increase the Pretreat digestion time/ intensity - Reduce the fixation time - Adjust incubation time of Amp solutions |
|  | - Non-specific binding of target probes | - Increase the hybridization temperature (up to 50ºC) - Increase the number of washes and washing time while subjecting the tubes to very slow agitation - Lay the tubes horizontally to allow efficient washing - Reduce the amount of the probe and/or the hybridization time |
| **No signal in one of the channels** | - One of the target probes is missing in the hybridization mix | - Recheck adding target probes to the hybridization mixture |
|  | - Insufficient amount of the target probe used | - Increase the amount of the probe used in the mixture |
|  | - Precipitated target probe | - Preheat target probes to 40°C before preparation of the target mix |
|  | - Conditional (stage or tissue specific) or no gene expression | - Check available gene expression databases for locus and stage specific expression, use PCR to check for RNA expression, or check for the expression pattern using conventional WISH |
|  | - Wrong target sequence provided | - Recheck the ordered target sequence |
|  | - Microscope settings for corresponding fluorophore not adjusted (wavelength etc.) | - Adjust microscope settings for the corresponding fluorophore (a channel assessment slide from ACD available) |
| **No signal in all of the channels** | - Hybridization temperature too high | - Decrease hybridization temperature (down to 40ºC) |
|  | - Detection kit used in the wrong order | - Use Amp solutions in the correct order |
|  | - Incorrect amplification temperature | - Adjust temperature during the amplification to 40°C |
|  | - Inadequate probe hybridization time | - Prolong the probe hybridization time |
| **Non-homogeneous signal** | - Heterogeneous accessibility of the embryos to the solution at the bottom of the tube | - Lay the tubes horizontally, while subjecting the tubes to very slow agitation |
| **No optimal Counterstaining** | - DAPI/ Hoechst stain did not fully penetrate the tissue | - Increase the DAPI/ Hoechst incubation time |
